# IgG hexamers initiate complement-dependent acute lung injury

**DOI:** 10.1172/JCI178351

**Published:** 2024-03-26

**Authors:** Simon J. Cleary, Yurim Seo, Jennifer J. Tian, Nicholas Kwaan, David P. Bulkley, Arthur E.H. Bentlage, Gestur Vidarsson, Éric Boilard, Rolf Spirig, James C. Zimring, Mark R. Looney

**Affiliations:** 1 Department of Medicine and; 2 Department of Biochemistry and Biophysics, UCSF, San Francisco, California, USA.; 3 Sanquin Research, Amsterdam, Netherlands.; 4 Centre de Recherche du Centre Hospitalier Universitaire de Québec – Université Laval, Québec, Quebec, Canada.; 5 CSL Behring, Research, CSL Behring Biologics Research Center, Bern, Switzerland.; 6 Department of Pathology, University of Virginia School of Medicine, Charlottesville, Virginia, USA.

**Keywords:** Pulmonology, Transplantation, Antigen, Immunoglobulins, Innate immunity

## Abstract

Antibodies can initiate lung injury in a variety of disease states such as autoimmunity, in reactions to transfusions, or after organ transplantation, but the key factors determining in vivo pathogenicity of injury-inducing antibodies are unclear. Harmful antibodies often activate the complement cascade. A model for how IgG antibodies trigger complement activation involves interactions between IgG Fc domains driving the assembly of IgG hexamer structures that activate C1 complexes. The importance of IgG hexamers in initiating injury responses was not clear, so we tested their relevance in a mouse model of alloantibody- and complement-mediated acute lung injury. We used 3 approaches to block alloantibody hexamerization (antibody carbamylation, the K439E Fc mutation, or treatment with domain B from staphylococcal protein A), all of which reduced acute lung injury. Conversely, Fc mutations promoting spontaneous hexamerization made a harmful alloantibody into a more potent inducer of acute lung injury and rendered an innocuous alloantibody pathogenic. Treatment with a recombinant Fc hexamer “decoy” therapeutic protected mice from lung injury, including in a model with transgenic human FCGR2A expression that exacerbated pathology. These results indicate an in vivo role of IgG hexamerization in initiating acute lung injury and the potential for therapeutics that inhibit or mimic hexamerization to treat antibody-mediated diseases.

## Abstract 2

Alloreactive IgG antibodies can form hexamers to trigger acute lung injury in mouse models.

## Graphical abstract

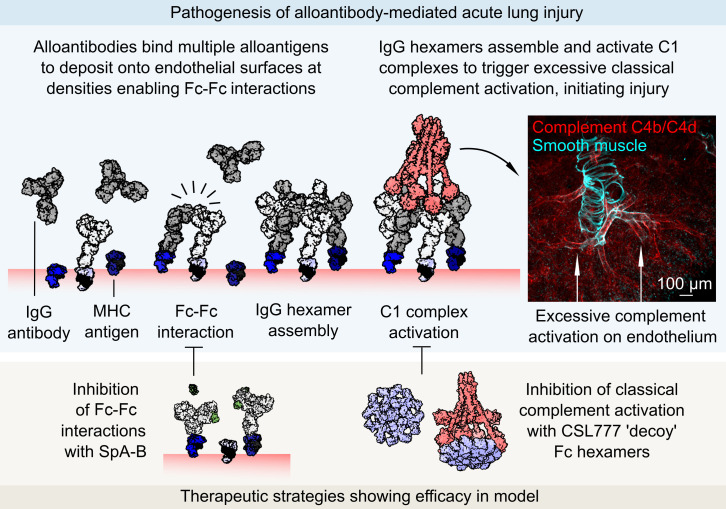

## Introduction

Antibodies and the complement cascade mediate protective immunity but can both become misdirected to cause harm in autoimmune and alloimmune diseases. Some antibodies direct activation of the complement cascade at their targets, an event associated with severe pathology in disease states including several forms of transfusion reactions (1, 2), immune rejection after solid organ transplantation (3), and complications of pregnancy (4). Complement-activating alloantibodies are known mediators of transfusion-related acute lung injury (TRALI) (5, 6), a leading cause of transfusion-related deaths (7), and are linked to particularly poor outcomes following solid organ transplantation (3, 8). Complement activation by autoreactive antibodies also contributes to pathogenesis of forms of autoimmune hemolytic anemia (9), small-vessel vasculitis (10), and neurological autoimmune disease (11).

IgG antibodies are the most prevalent type of antibody in circulation, and complement-activating alloantibodies are frequently of the IgG class. IgG antibodies achieve complement activation through recruitment and activation of C1 complexes, each of which contains 6 Fc-binding domains (12, 13). A theory for how IgG achieves C1 complex activation involves groups of 6 IgG antibodies interacting through their Fc domains to form IgG hexamers (14). This theory recently gained experimental support from direct imaging of IgG1 and IgG3 hexamer assembly on antigenic liposomes (15, 16), with in vitro studies connecting IgG hexamerization to increased complement deposition on target surfaces (15, 17, 18). However, it is currently unclear whether IgG hexamer assembly is important in vivo in the pathogenesis of complement-dependent forms of alloantibody-mediated disease.

Here, we report testing of interventions exploiting IgG hexamerization in a mouse model of acute lung injury driven by alloantibody deposition in the pulmonary microvasculature, a process that drives pathology in forms of both TRALI and antibody-mediated rejection (AbMR) of lung transplants (6). Our results identify key molecular events driving alloantibody-mediated pathophysiology in vivo. We also demonstrate preclinical efficacy of new therapeutic approaches preventing complement-dependent organ damage caused by alloantibodies, serving as a rationale to pursue translational studies in human alloantibody–driven disease.

## Results

Alloantibodies are prevalent but not always harmful, so determining whether alloantibodies are clinically significant is a frequent conundrum in transfusion and transplantation medicine. Reflecting this clinical challenge, of the many mouse monoclonal alloantibodies targeting MHC class I antigens, only clone 34-1-2S triggers acute lung injury when microgram quantities are i.v. injected into mice (19, 20). In addition, only mice expressing the H-2^d^ MHC class I haplotype are known to be susceptible to injury caused by the 34-1-2S antibody (5, 6). Curiously, the 34-1-2S antibody does not readily cause injury in H-2^b^ mice, including the widely used C57BL/6 (B6) strain, despite the fact that it binds to MHC class I antigens expressed by H-2^b^ mice (5, 6). We aimed to improve our understanding of the factors determining the ability of antibodies to cause injury both in this widely used model and more generally in antibody-mediated disease states.

**Figure 1:**
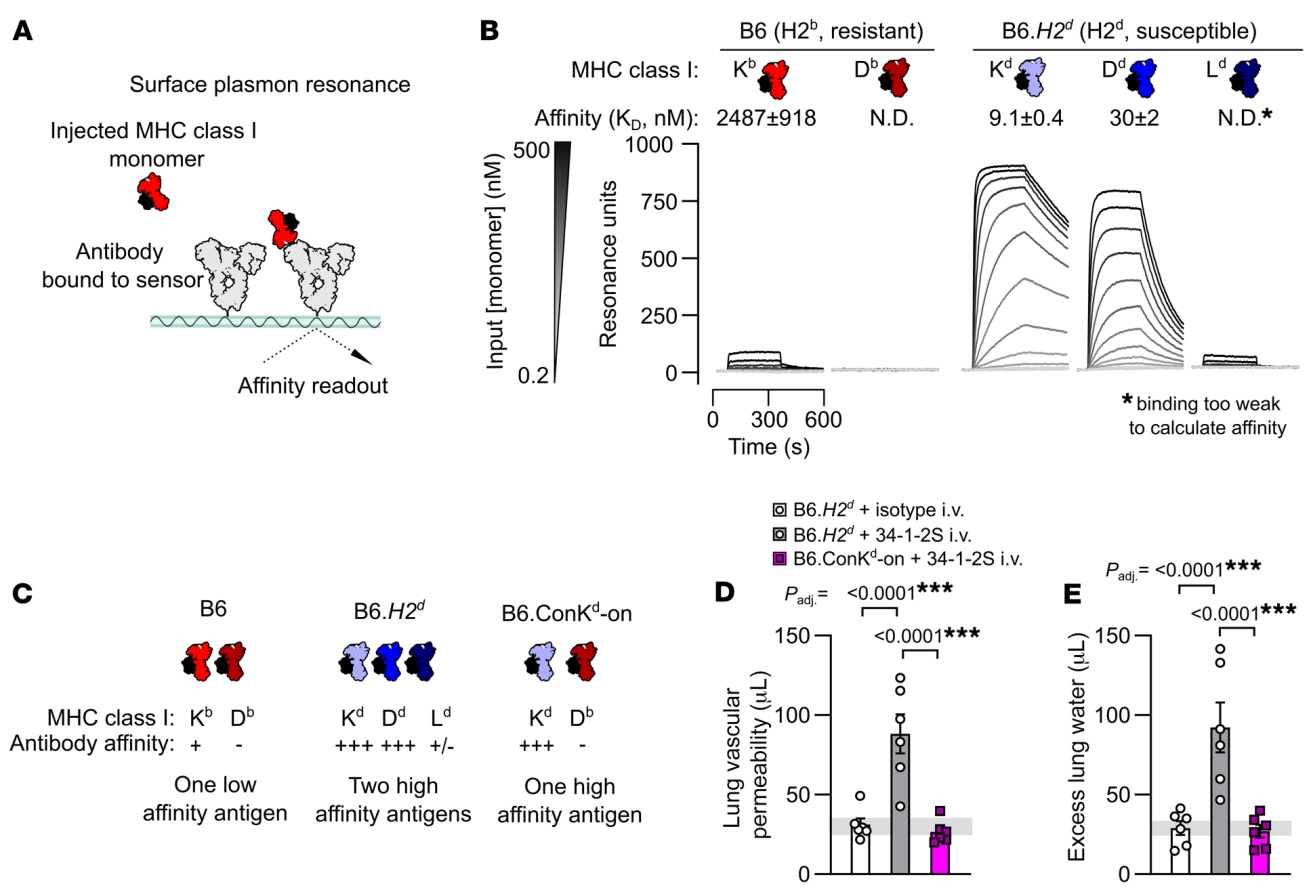
The 34-1-2S alloantibody binds to multiple MHC class I antigens to trigger acute lung injury. (**A**) Schematic showing the approach for measuring affinity of the MHC class I alloantibody 34-1-2S for MHC class I monomers using SPR. (**B**). SPR sensorgrams showing detection of the binding of 34-1-2S to the K^b^ MHC class I antigen from H2^b^ mice and the K^d^, D^d^, and L^d^ antigens from mice with the H2^d^ haplotype. (**C**) Classical MHC class I antigens present in B6, B6.*H2^d^* and B6.Con-K^d^-on mice with a summary of the results from **B**. (**D**) Lung vascular permeability and (**E**) excess lung water measurements from LPS-primed B6.*H2^d^* mice given i.v. doses of either 34-1-2S or isotype control versus B6.Con-K^d^-on mice given i.v. 34-1-2S. Depictions of IgG and MHC class I in **A**–**C** are based on the Protein Data Bank (PDB) entries 1HZH and 1RK1. Data in **B**, **D** and **E** indicate the mean ± SEM. *P* values adjusted for multiple comparisons (*P*_adj._) in **D** and **E** were determined by ordinary 1-way ANOVA with Dunnett’s test for differences relative to the B6.*H2^d^* plus 34-1-2S i.v. group, with data being log_10_-transformed prior to analysis. ***P < 0.0001.

We measured the binding affinity of 34-1-2S antibody to each of the classical MHC class I antigens present in injury-resistant H-2^b^ B6 mice and injury-susceptible H-2^d^ mice (Figure 1A). Of the 3 MHC class I antigens in the H-2 locus (K, D, and L), B6 mice only express K^b^ and D^b^, and we detected binding of 34-1-2S to K^b^ but not D^b^. In contrast, we detected binding of 34-1-2S to all 3 MHC class I antigens from H-2^d^ mice, with high-affinity binding to K^d^ and D^d^ and weak binding to L^d^ (Figure 1B). Other MHC class I antibodies (clones AF6-88.5.5.3, 20-8-4S, SF1.1.10, 30-5-7S and 34-5-8S), which do not readily induce injury (5), each bound to only 1 MHC class I antigen from each MHC type (Supplemental Figure 1; supplemental material available online with this article; https://doi.org/10.1172/JCI178351DS1).

Together, the above findings led us to the hypothesis that the ability of 34-1-2S to induce lung injury in H-2^d^ mice is a function of increased density of bound antibody in H-2^d^ animals, resulting from simultaneous binding of 34-1-2S to K^d^, D^d^, and possibly L^d^. This hypothesis was tested by injecting 34-1-2S antibody into B6.ConK^d^-on mice, which express K^d^ but do not express D^d^ or L^d^ (21). B6.*H2^d^* mice expressing the full complement of MHC class I antigens recognized by 34-1-2S (K^d^, D^d^, and L^d^) were used as background-matched positive controls for susceptibility to injury (Figure 1C). In contrast to B6.*H2^d^* mice, B6.ConK^d^-on mice did not develop lung injury (Figure 1, D and E). These data are consistent with 34-1-2S antibody causing injury in H2^d^ mice through high-affinity binding to multiple MHC class I antigens.

**Figure 2:**
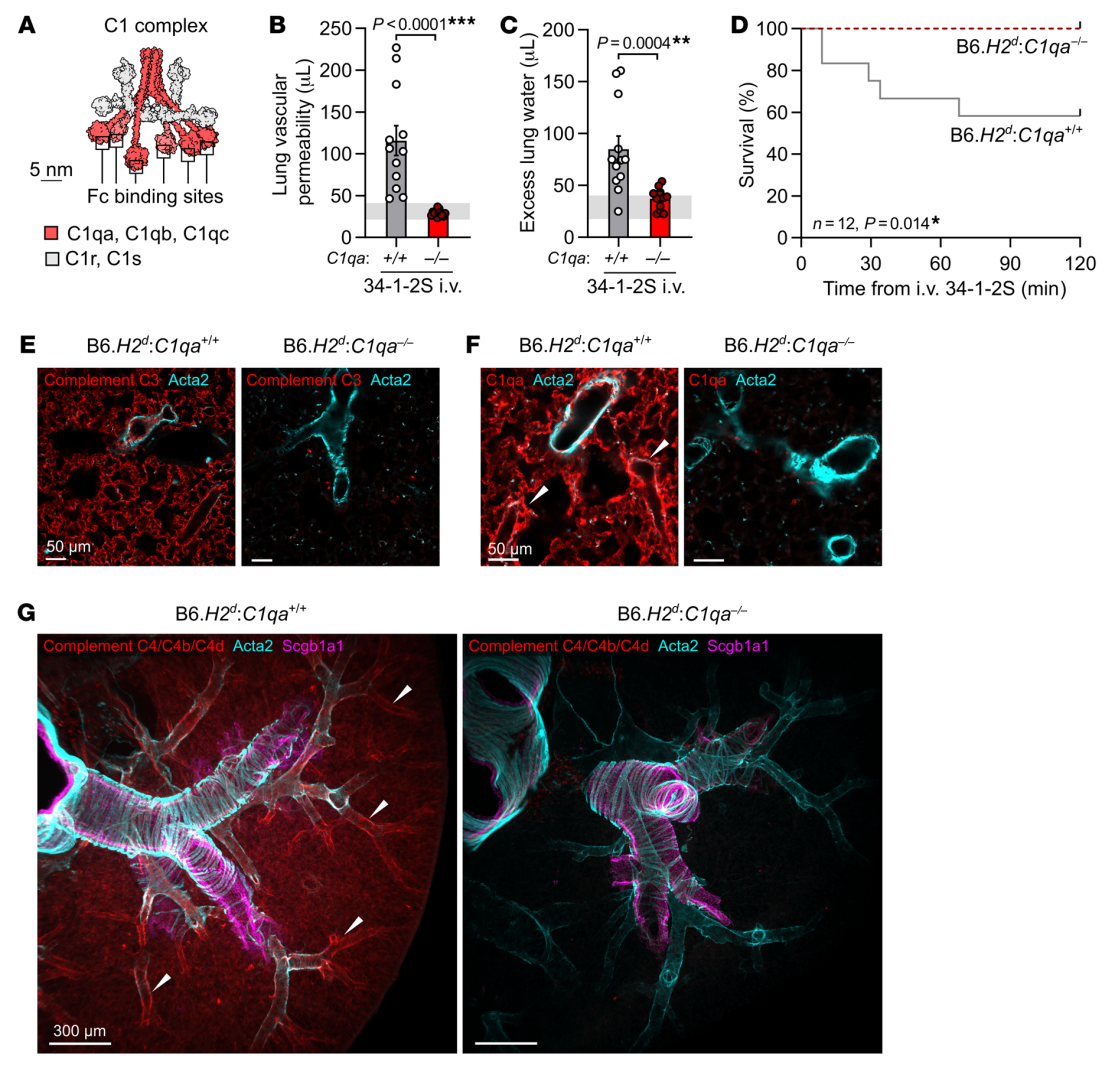
Classical complement activation on the pulmonary endothelium initiates 34-1-2S–induced acute lung injury. (**A**) Molecular model of the C1 complex based on the small-angle scattering database entry SASDB38 (12). (**B**) Lung vascular permeability and (**C**) excess lung water measurements from LPS-primed B6.*H2^d^* *C1qa^–/–^* mice and B6.*H2^d^* *C1qa^+/+^* littermates given i.v. 34-1-2S at 1 mg/kg. Horizontal gray lines are SDs of values from “no-injury” controls (B6.*H2^d^* mice given LPS i.p. plus mIgG2a isotype control i.v.). (**D**) Survival of LPS-primed B6.*H2^d^* *C1qa^–/–^* mice and B6.*H2^d^* *C1qa^+/+^* littermates given i.v. 34-1-2S at 4.5 mg/kg. (**E**–**G**) Immunofluorescence staining for (**E**) complement C3, (**F**) C1qa, and (**G**) C4/C4b/C4d (red) as well as for α–smooth muscle actin (Acta2, cyan) and Scgb1a1 (club cell secretory protein, magenta) in lung sections from LPS-primed B6.*H2^d^* *C1qa^–/–^* mice and B6.*H2^d^* *C1qa^+/+^* mice fixed 5 minutes after i.v. injection of 1 mg/kg 34-1-2S. Images in **G** are maximum-intensity projections sampling 240 μm from a cleared thick section. White arrowheads point to arterioles positive for complement components. Scale bars: 50 um (**E** and **F**); 300 μm (**G**). Data in **B** and **C** show the mean ± SEM. **P < 0.01 and ***P < 0.0001, by unpaired, 2-tailed *t* test on log_10_-transformed data (**B** and **C**) or log-rank test (**D**). *n* = 12/group; *n* = 3 samples/group for immunofluorescence staining.

Engagement of multiple antigens can permit high-density antibody deposition, an event associated with classical complement activation. Complement activation has been implicated in the pathogenesis of acute lung injury caused by 34-1-2S antibody, but previous studies have not determined whether injury in this model is directly triggered by antibody-mediated complement activation via the classical pathway (5, 6). To test whether 34-1-2S–induced injury requires classical complement activation, we bred mice expressing the *H2^d^* susceptibility locus with mice lacking C1qa (22), a protein that is necessary for classical complement activation, as it is 1 of the 3 proteins that make up each of the 6 Fc-binding C1q subcomponents in each C1 complex (Figure 2A).

Relative to B6.*H2^d^* *C1qa^+/+^* littermate controls, C1qa-deficient B6.*H2^d^* *C1qa^–/–^* mice were resistant to alloantibody-mediated acute lung injury and mortality (Figure 2, B–D). Mice lacking C1qa were also protected from deposition of complement component C3 split products on the endothelium of pulmonary capillaries (Figure 2E). Staining for C1qa in lungs confirmed an absence of C1qa protein in KO mice, with intense C1 complex deposition seen around pulmonary arterioles in C1qa-expressing mice injected with 34-1-2S antibody (Figure 2F).

To identify the microanatomical site of classical complement activation, we stained lungs of mice injected with 34-1-2S for the complement C4 split products C4b and C4d, which form covalent bonds with proteins at sites of classical complement activation. We observed strong positivity for C4b/d, highlighting the endothelium of medium- and small-sized pulmonary arterioles in B6.*H2^d^* *C1qa^+/+^* mice injected with 34-1-2S, but not in B6.*H2^d^* *C1qa^–/–^* mice (Figure 2G and Supplemental Video 1). Together, these results indicate that 34-1-2S caused acute lung injury because this antibody was deposited onto the pulmonary arteriolar endothelium at densities sufficient to trigger excessive classical complement activation directed at the walls of these blood vessels.

**Figure 3:**
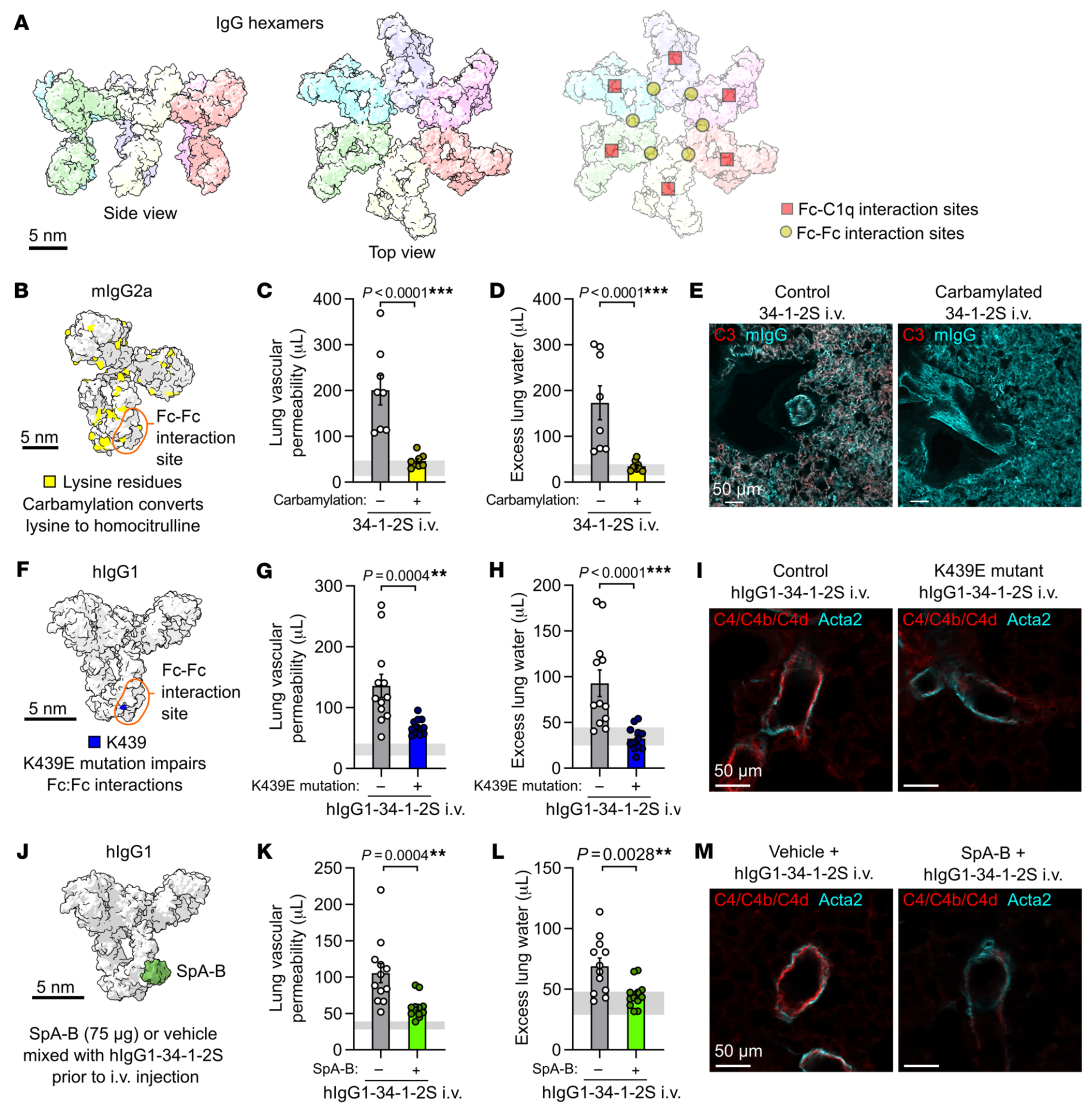
Inhibiting IgG hexamer assembly reduces 34-1-2S-induced acute lung injury responses. (**A**) Molecular models of IgG hexamers based on the PDB entry 1HZH, showing Fc-Fc and Fc-C1q interaction sites. Scale bar: 5 nm. (**B**) Molecular model showing lysine residues in mouse IgG2a (mIgG2a), per PDB entry 1IGT. (**C**) Lung vascular permeability, (**D**) excess lung water measurements, and (**E**) lung complement C3 and mIgG immunostains from LPS-primed BALB/c mice after i.v. injection of carbamylated or control 34-1-2S. (**F**) Molecular model showing the location of Fc domain lysine 439 (K439) in hIgG1, per PDB entry 1HZH. (**G**) Lung vascular permeability, (**H**) excess lung water measurements, and (**I**) lung complement C4/C4b/C4d and Acta2 immunostains from LPS-primed B6.*H2^d^* mice after i.v. injection with hIgG1-34-1-2S or hIgG1-34-1-2S with the K439E mutation.(**J**) Molecular model showing the binding site of SpA-B to the Fc domain of hIgG1, per the PDB entries 1HZH and 5U4Y. (**K**) Lung vascular permeability, (**L**) excess lung water measurements, and (**M**) lung complement C4/C4b/C4d and Acta2 immunostains from LPS-primed B6.*H2^d^* mice after i.v. injection with hIgG1-34-1-2S and 75 μg SpA-B or vehicle control. Samples for the injury measurements were collected 2 hours after antibody injections, and lungs were fixed for immunostaining 5 minutes after antibody injections. Graphs show the mean ± SEM, with horizontal gray lines showing 95% CIs of measurements from no-injury control mice given LPS and nonreactive isotype antibodies. **P < 0.01 and ***P < 0.0001, by unpaired, 2-tailed *t* tests on log_10_-transformed data, with *n* = 8/group (**C** and **D**) and *n* = 12/group (**G**, **H**, **K**, and **L**), or are representative of 3 samples/group (**E**, **I**, and **M**). Scale bars: 50 μm (**E**, **I**, and **M**).

Dense binding to membrane-expressed antigens would be expected to facilitate IgG Fc-Fc interactions and IgG hexamer assembly. IgG hexamers are potent activators of C1 complexes in vitro (15) and are further implicated in classical complement activation by models for C1 complex activation involving shifting of its 6 Fc-binding C1q subcomponents into a regular hexagonal configuration (Figure 2A and Figure 3A) (12, 13, 23). We therefore hypothesized that 34-1-2S assembles into hexamers on the pulmonary endothelial surface of susceptible mice to trigger complement-dependent acute lung injury.

Imaging methods cannot currently resolve IgG hexamers in vivo, but recent studies have developed methods for inhibiting IgG hexamerization. One approach to impair IgG hexamer assembly is to carbamylate antibodies, converting lysine residues to homocitrullines to alter charge densities in IgG Fc regions, inhibiting Fc-Fc interactions and IgG hexamer assembly (Figure 3B) (24). Mice treated with carbamylated 34-1-2S showed greatly reduced acute lung injury responses compared with littermate controls treated with noncarbamylated 34-1-2S (Figure 3, C and D, and Supplemental Figure 2, A–D). Carbamylated 34-1-2S retained its ability to bind antigens and become deposited in lungs but, in contrast to unchanged 34-1-2S, did not induce complement C3b/d deposition in the pulmonary microvasculature (Figure 3E and Supplemental Figure 2, E and F)

Lysine residues are present on regions of IgG outside of the Fc-Fc interaction interface (illustrated in Figure 3B), including at the Fc-C1q interaction site (25), so we pursued a more targeted strategy for inhibition of IgG hexamer assembly. We determined the sequence of both heavy- and light-chain complementarity–determining regions and engineered a chimeric antibody with the Fab domain of 34-1-2S fused in-frame to human IgG1 (hIgG1-34-1-2S). To test whether hIgG1-34-1-2S causes injury through hexamerization, we also expressed this antibody with an Fc point mutation that inhibits Fc-Fc interactions required for IgG hexamer assembly (K439E) (Figure 3F) (15). Like mouse IgG2a 34-1-2S, hIgG1-34-1-2S injections caused acute lung injury (Figure 3, G and H, and Supplemental Figure 3, A and B). Lung vascular permeability and pulmonary edema responses were reduced by the K439E mutation (Figure 3, G and H), as was complement C4b/d deposition in lungs (Figure 3I), lending further support to a role for Fc-Fc interactions and hexamerization in the pathogenesis of this disease model.

We also tested a strategy for pharmacologic inhibition of Fc-Fc interactions by mixing hIgG1-34-1-2S with recombinant B domains from *Staphylococcus aureus* protein A (SpA-B), which bind to IgG antibodies near Fc-Fc interaction sites and inhibit hexamer assembly and complement activation by antibodies targeting bacterial antigens (Figure 3J) (17, 26). We hypothesized that these properties of SpA-B, which likely evolved as part of an immune evasion strategy, might be harnessed to prevent hIgG1-34-1-2S from causing acute lung injury. Adding SpA-B to hIgG1-34-1-2S reduced its ability to both induce acute lung injury (Figure 3, K and L) and cause complement C4b/d deposition within pulmonary arterioles (Figure 3M). These findings provide a third line of evidence that Fc-Fc interactions leading to hexamer assembly are important for the injury response caused by this alloantibody.

**Figure 4:**
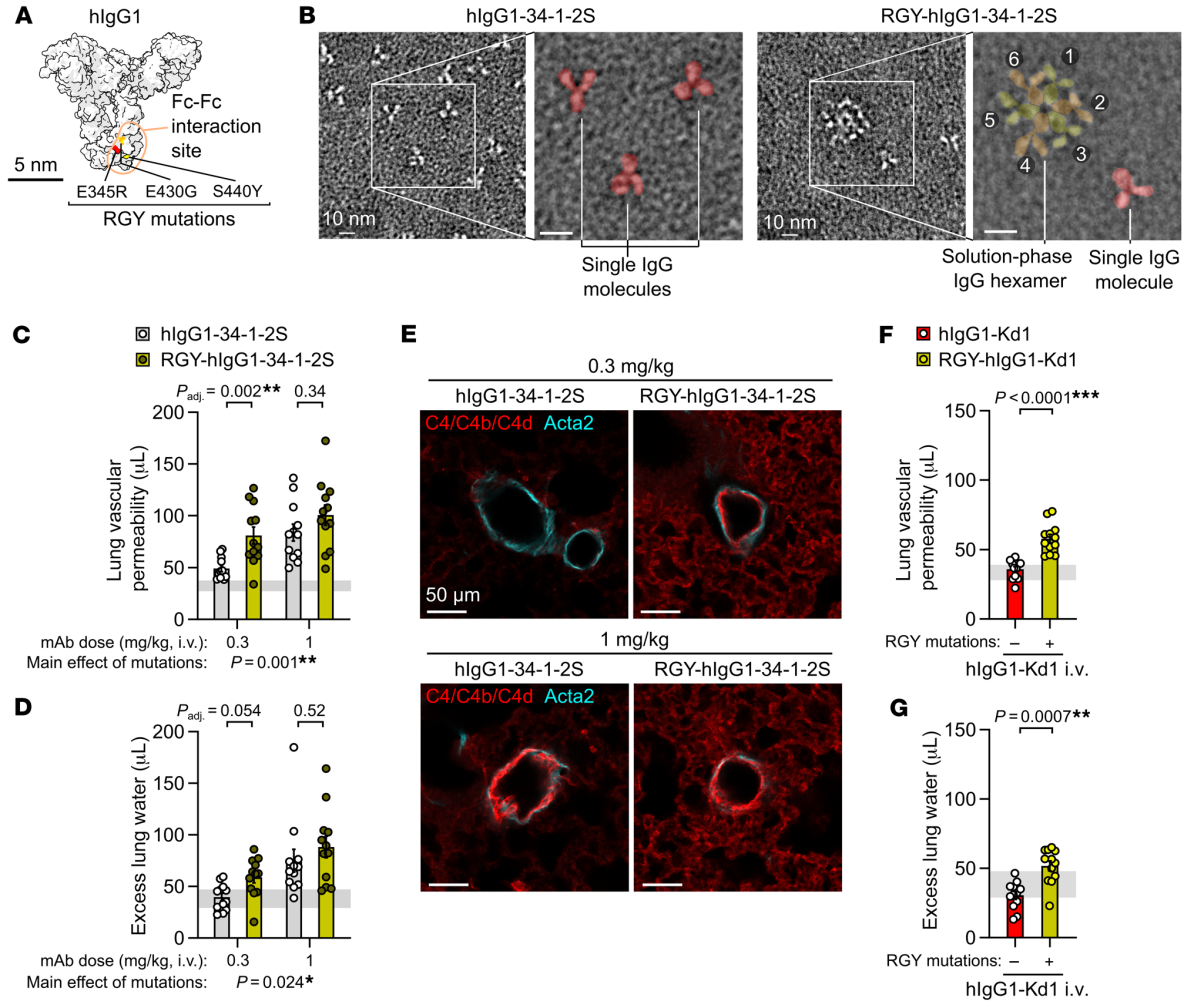
Fc mutations promoting IgG hexamer assembly increase the in vivo pathogenicity of alloantibodies. (**A**) Molecular model showing amino acids mutated in RGY-hIgG1 antibodies, per PDB entry 1HZH. (**B**) Negative stain electron micrographs showing single hIgG1-34-1-2S molecules and spontaneous solution-phase hexamers formed by RGY-hIgG1-34-1-2S (colored overlay highlights structures in expanded images). Numbers 1–6 label individual IgG molecules forming a hexamer. Scale bars: 10 nm (including enlarged insets). (**C**) Lung vascular permeability and (**D**) excess lung water measurements from LPS-primed B6.*H2^d^* mice injected with control or RGY-mutated hIgG1-34-1-2S mAbs at i.v. doses of either 0.3 or 1 mg/kg. (**E**) Immunofluorescence staining showing pulmonary arterioles stained for complement C4/C4b/C4d (red) and Acta2 (cyan) in lung sections from LPS-primed B6.*H2^d^* mice given the indicated treatments, representative of 3 samples per group fixed 5 minutes after antibody injections. Scale bars: 50 μm. (**F**) Lung vascular permeability and (**G**) excess lung water measurements from LPS-primed B6.*H2^d^* mice injected with control or RGY-mutated hIgG1-Kd1 (a novel mAb targeting only the H-2K^d^ MHC class I antigen) at 1 mg/kg i.v. Graphs show the mean ± SEM, with the horizontal line representing 95% CIs from no-injury controls (LPS-primed B6.*H2^d^* mice given hIgG1 isotype control i.v.). *P < 0.05, **P < 0.01 and ***P < 0.0001; log_10_-transformed data were analyzed using an ordinary 2-way ANOVA with Šídák’s multiple-comparison test for the effect of the Fc mutation within the dose level (**C** and **D**) or unpaired, 2-tailed *t* test (**F** and **G**). *n* = 12/group.

Turning to hexamer gain-of-function experiments, the introduction of 3 mutations into the Fc domain of hIgG1 (RGY mutations: E345R, E430G, S440Y) has yielded antibodies capable of off-target hexamer assembly as well as increased on-target hexamerization (15) (Figure 4A). We hypothesized that RGY-mutated 34-1-2S (RGY-hIgG1-34-1-2S) would have an enhanced ability to cause acute lung injury due to increased IgG hexamer formation. We produced RGY-hIgG1-34-1-2S and confirmed its ability to spontaneously assemble into hexamers in solution (Figure 4B). Consistent with a role for alloantibody hexamerization in driving injury, RGY-hIgG1-34-1-2S showed increased potency in triggering acute lung injury relative to hIgG1-34-1-2S (Figure 4, C and D). It is likely that, because of the ability of hIgG1-34-1-2S to form hexamers and cause complement-dependent injury when injected at 1 mg/kg, the effects of the RGY mutations were only apparent at a lower dose (0.3 mg/kg), at which unaltered hIgG1-34-1-2S did not provoke complement C4b/d deposition in lungs (Figure 4E). Consistent with the fact that binding to multiple antigens is required for alloantibody-mediated acute lung injury, a novel chimeric hIgG1 antibody binding a single MHC class I antigen (hIgG1-Kd1, targeting K^d^) did not provoke injury when injected into B6.*H2^d^* mice (Figure 4, F and G). However, introduction of the RGY mutations promoting hexamerization into this innocuous antibody resulted in a modified version (RGY-hIgG1-Kd1) that was able to provoke increases in lung vascular permeability and edema (Figure 4, F and G). These findings demonstrate that mutations promoting IgG hexamer assembly can increase the pathogenicity of antibodies at doses that do not normally result in sufficiently dense binding to trigger complement-dependent injury responses.

Another approach for therapeutic exploitation of IgG hexamerization involves the use of Fc hexamers as “decoy” treatments. These therapeutic candidates are being studied as recombinant alternatives to plasma-derived intravenous or subcutaneous immunoglobulin (IVIg or SCIg) treatments that are used in the management of autoimmune and alloimmune diseases (27). We hypothesized that, because of its ability to inhibit classical complement activation (11, 28), the Fc hexamer decoy treatment CSL777 (previously Fc-μTP-L309C) would be effective in preventing alloantibody-mediated acute lung injury.

**Figure 5:**
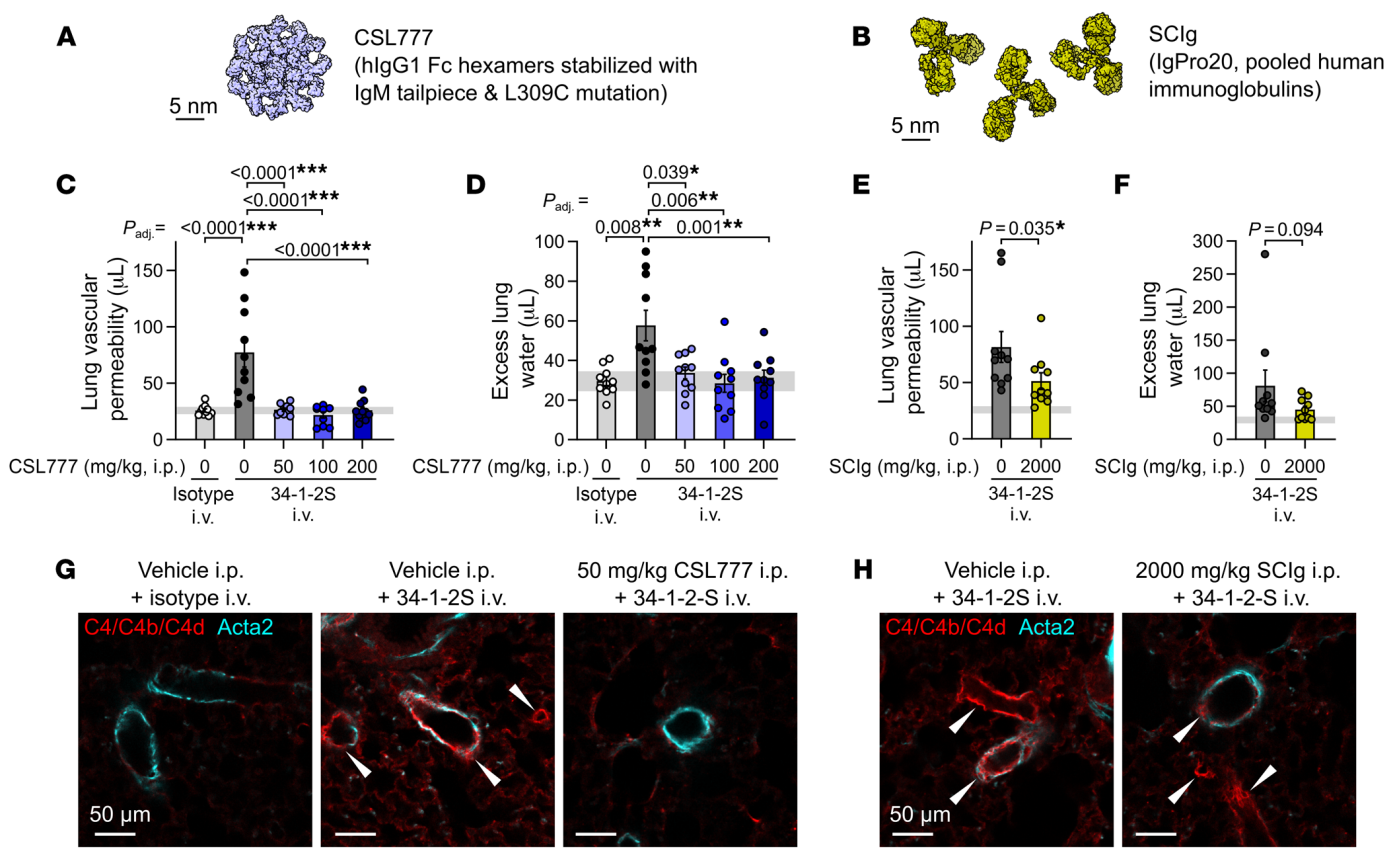
Treatment with recombinant Fc hexamer decoys prevents alloantibody-mediated acute lung injury. (**A**) Molecular representation of CSL777, an investigational recombinant Fc hexamer decoy treatment that inhibits classical complement activation, based on PDB entry 7X13 (52). (**B**) Molecular representation of SCIg, a pooled hIg therapeutic with antiinflammatory properties at high doses, based on PDB entry 1HZH. Scale bar: 5 nm. (**C**) Lung vascular permeability and (**D**) excess lung water measurements from LPS-primed BALB/c mice given i.p. vehicle or CSL777 at the indicated doses 1 hour before i.v. injection of 34-1-2S or mIgG2a isotype control. (**E**) Lung vascular permeability and (**F**) excess lung water measurements from LPS-primed BALB/c mice given i.p. vehicle or 2,000 mg/kg SCIg 1 hour before i.v. injection of 34-1-2S or mIgG2a isotype control. (**G** and **H**) Immunofluorescence showing pulmonary arterioles stained for complement C4/C4b/C4d (red) and Acta2 (cyan) in lung sections from LPS-primed BALB/c mice given the indicated treatments, representative of 3 samples per group fixed 5 minutes after antibody injections. White arrowheads point to arterioles with endothelial positivity for C4/C4b/C4d. Scale bars: 50 μm. Graphs show the mean ± SEM, with the horizontal line representing 95% CIs of data from no-injury controls (from vehicle plus isotype control group). *P < 0.05, **P < 0.01 and ***P < 0.0001; log_10_-transformed data were analyzed using either (**C** and **D**) an ordinary 1-way ANOVA (**C** and **D**), Dunnett’s multiple-comparison test for differences relative to the vehicle plus 34-1-2S group (**C** and **D**), or 2-tailed, unpaired *t* test (**E** and **F**). *n* = 10/group.

We randomized mice to receive either CSL777, SCIg (IgPro20, a human plasma–derived Ig product that is currently used to treat antibody-mediated diseases), or relevant vehicle controls prior to injection with 34-1-2S (Figure 5, A and B). Treatment with CSL777 protected mice from developing 34-1-2S–induced lung vascular permeability and pulmonary edema responses at all doses tested, whereas treatment with SCIg only had a partial effect on alloantibody-induced acute lung injury responses (Figure 5, C–F). CSL777-treated mice lacked alloantibody-mediated deposition of complement C4 split products on pulmonary arterioles, whereas arteriolar endothelial C4b/d deposition was still observed in the lungs of SCIg-treated mice after 34-1-2S antibody injections (Figure 5, G and H). Recombinant Fc hexamer therapeutics such as CSL777 might therefore be useful for the prevention or treatment of complement-dependent forms of alloantibody-mediated organ injury.

**Figure 6:**
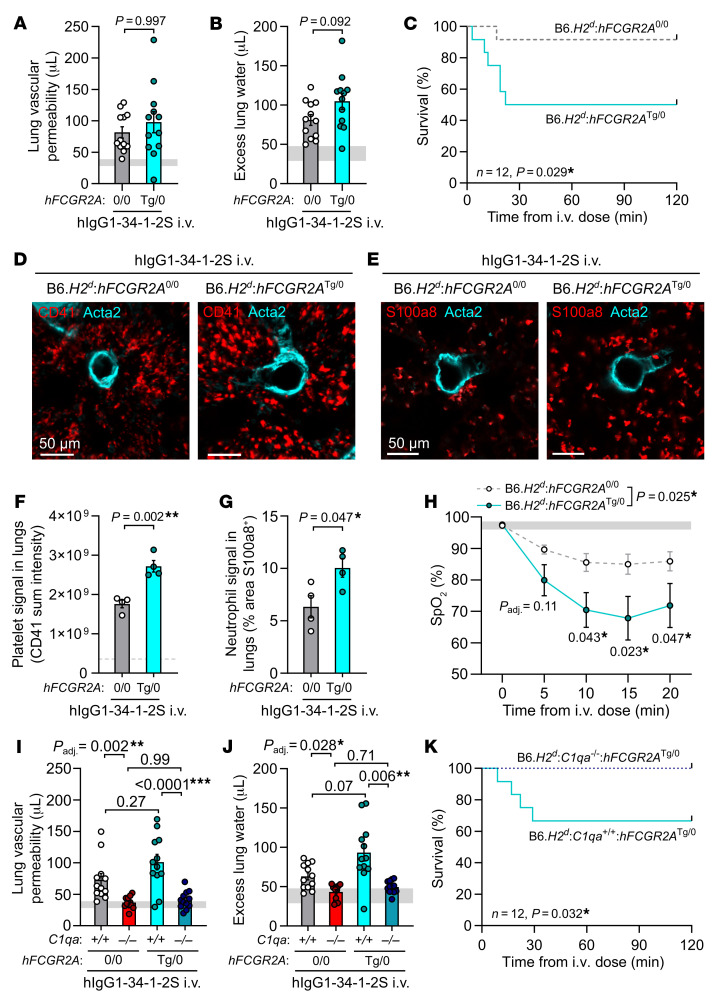
Acute lung injury is complement dependent in a model incorporating human FCGR2A–mediated pathology. (**A**) Lung vascular permeability, (**B**) excess lung water, and (**C**) survival readouts from LPS-primed B6.*H2^d^*:*hFCGR2a*^Tg/0^ mice and littermate controls lacking hFCGR2A expression that were given i.v. hIgG1-34-1-2S at 1 mg/kg. (**D**) Immunofluorescence images of platelet sequestration (CD41, red, with Acta2 in cyan) and (**E**) neutrophils (S100a8, red, with Acta2 in cyan) in lungs from LPS-primed B6.*H2^d^*:*hFCGR2a*^Tg/0^ mice and littermates without hFCGR2A expression, fixed at 20 minutes after hIgG1-34-1-2S injections and (**F** and **G**) quantification. Scale bars: 50 μm. (**H**) SpO_2_ measurements from LPS-primed B6.*H2^d^*:*hFCGR2a*^Tg/0^ mice and littermate controls without hFCGR2A expression before and after hIgG1-34-1-2S injections. (**I**) Lung vascular permeability, (**J**) excess lung water, and (**K**) survival readouts for LPS-primed B6.*H2^d^* *C1qa^+/+^* and B6.*H2^d^* *C1qa^–/–^* mice, as well as for hFCGR2A-expressing littermates of each genotype, that were given i.v. hIgG1-34-1-2S at 1 mg/kg. Data in **A**, **B**, **F**, **G**, **H**, **I**, and **J** show the mean ± SEM, with horizontal gray lines showing values from the no-injury controls (baseline readings or values from B6.*H2^d^* mice given LPS i.p. plus hIgG1 isotype control i.v.), and were log_10_ transformed prior to analysis. *P < 0.05, **P < 0.01 and ***P < 0.0001, by unpaired, 2-tailed *t* tests (**A**, **B**, **F**, and **G**); 2-way ANOVA with Šídák’s multiple-comparison test (**I** and **J**); log-rank test (**C** and **K**); or 2-way, repeated-measures mixed-model approach with tests for main effect of the genotype and for post-baseline effects of the genotype within time levels with Holm’s adjustment for multiple comparisons (**H**). *n* = 4/group (**D**–**G**); *n* = 10/group (**H**); *n* = 12/group (other graphs).

Unlike humans, mice do not express the Fcγ receptor FCGR2A (also known as FcγRIIA or CD32A), negatively affecting the predictive value of mouse models for studying human antibody–mediated diseases (29, 30). To test whether our previous findings held up in a system involving FCGR2A-driven pathology, we crossed existing mouse lines to generate 34-1-2S–mediated injury–susceptible H-2^d^ mice expressing a human FCGR2A (hFCGR2A) transgene (B6.*H2^d^*:*hFCGR2A*^Tg/0^). In response to hIgG1-34-1-2S injections, mice expressing hFCGR2A developed similar degrees of lung injury relative to that seen in littermates without hFCGR2A expression but showed a survival disadvantage (Figure 6, A–C). B6.*H2^d^*:*hFCGR2A*^Tg/0^ mice expressed hFCGR2A on platelets, became more thrombocytopenic than did their littermates lacking hFCGR2A after hIgG1-34-1-2S injections, and occasionally died before the onset of pulmonary edema (Supplemental Figure 3, A–G). On the basis of these observations, we hypothesized that hFCGR2A expression enhances intravascular immunothrombotic responses that occur within 5–10 minutes of 34-1-2S injections (6), causing fatal hypoxic respiratory failure by impairing lung microvascular perfusion and increasing alveolar dead space. Supporting this hypothesis, within 20 minutes of hIgG1-34-1-2S injections, hFCGR2A transgenic mice had increased sequestration of platelets and neutrophils in their pulmonary microvasculature (Figure 6, D–G) and rapidly became more hypoxic than did littermates without hFCGR2A expression (Figure 6H). B6.*H2^d^*:*hFCGR2A*^Tg/0^ mice developing particularly severe hypoxemia (SpO_2_ readings <40%) did not survive until 2 hours after antibody injections (Supplemental Figure 3H). Surviving hFCGR2A transgenic mice went on to develop increased lung vascular permeability responses compared with littermates that did not express hFCGR2A, indicating that, given time to develop, responses to FCGR2A engagement also exacerbate pulmonary edema (Supplemental Figure 3, I and J), as recently described in a similar model (30). Classical complement activation was still critical for pathogenesis in the presence of hFCGR2A, as KO of *C1qa* protected hFCGR2A-expressing mice from lung injury and mortality (Figure 6, I–K).

**Figure 7:**
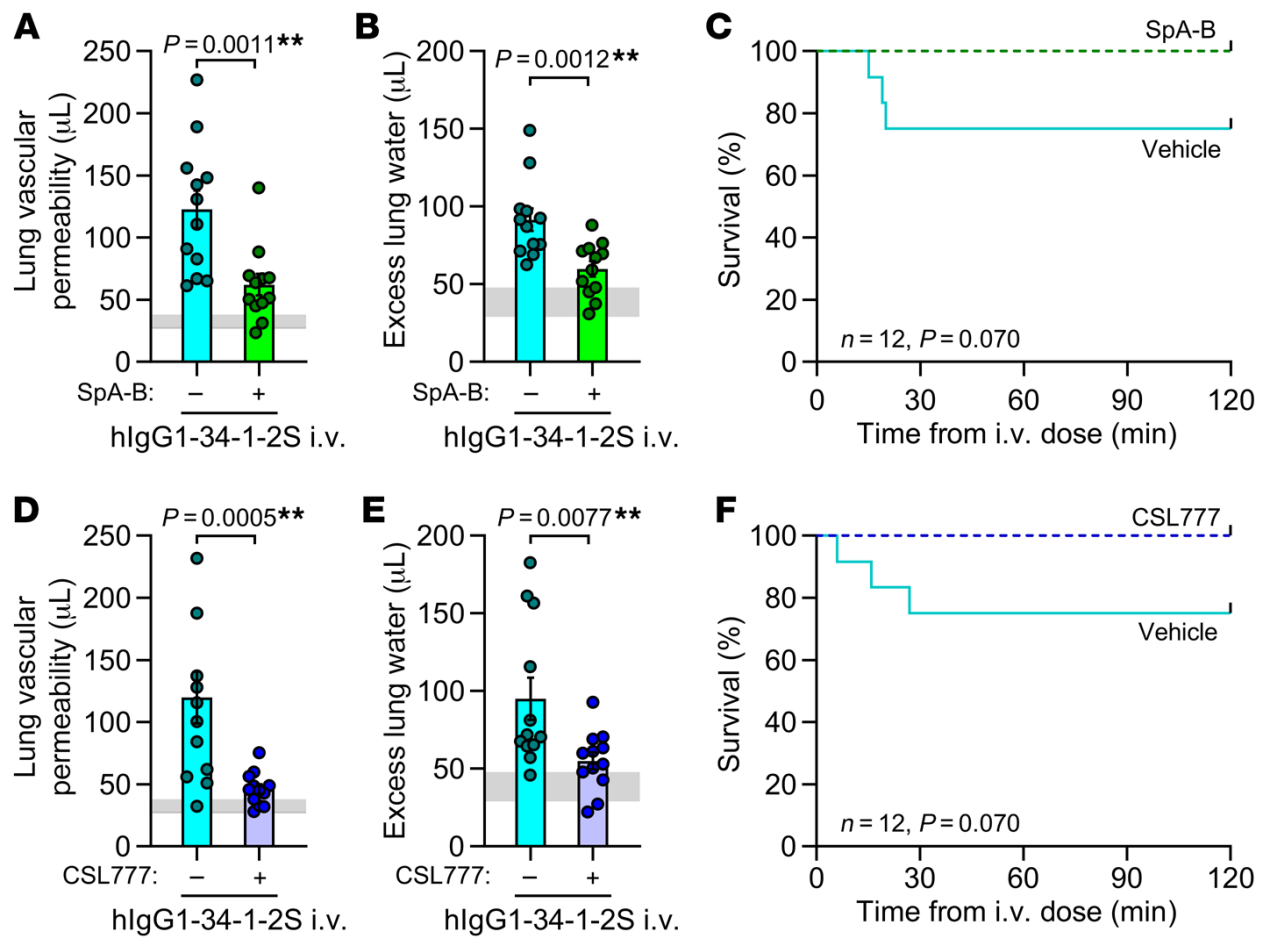
Approaches to inhibit or mimic IgG hexamerization reduce antibody-mediated acute lung injury in mice expressing human FCGR2A. (**A**) Lung vascular permeability, (**B**) excess lung water, and (**C**) survival readouts for LPS-primed B6.*H2^d^*:*hFCGR2a*^Tg/0^ mice given i.v. hIgG1-34-1-2S at 1 mg/kg with either the IgG hexamerization inhibitor SpA-B or vehicle. (**D**) Lung vascular permeability, (**E**) excess lung water, and (**F**) survival readouts for LPS-primed B6.*H2^d^*:*hFCGR2a*^Tg/0^ mice given either vehicle or 50 mg/kg CSL777 i.p. before i.v. injection of hIgG1-34-1-2S at 1 mg/kg. Data in **A**, **B**, **D**, and **E** show the mean ± SEM, with horizontal gray lines showing SDs of the values from the no-injury controls (B6.*H2^d^* mice given LPS i.p. plus hIgG1 isotype control i.v.) and were log_10_ transformed prior to analysis. **P < 0.01, by unpaired, 2-tailed *t* test (**A, B**, **D**, and **E**) or log-rank test (**C** and **F**) (all, *n* = 12/group).

Consistent with a retained importance of complement for initiation of injury responses in mice expressing FCGR2A, blocking hexamerization with SpA-B or pretreating mice with CSL777 hexamer decoys was protective in B6.*H2^d^*:*hFCGR2A*^Tg/0^ mice that were given hIgG1-34-1-2S injections (Figure 7, A–F). These results provide evidence that strategies to inhibit or mimic IgG hexamerization can maintain efficacy in the presence of increased severity of pathophysiology due to FCGR2A engagement.

## Discussion

This study advances our understanding of immunology in 3 areas. Our work elucidates the molecular determinants of susceptibility in a widely used inflammation model. To our knowledge, our experiments represent the first in vivo evidence that alloantibody hexamerization is important for pathophysiology. In addition, we show that 2 experimental therapeutic approaches that target antibody hexamerization can reduce alloantibody-mediated organ injury.

The initial aim or our study was to solve the mystery of why injections of 34-1-2S antibody (but not other monoclonal MHC class I antibodies) cause striking pathophysiology in H-2^d^ mice (but not in mice with other haplotypes) in what has become a widely used inflammation model (6, 19, 20, 30–40). Our results provide an explanation for this pattern of susceptibility: high-affinity binding to multiple MHC class I antigens on the pulmonary endothelium of mice with the H-2^d^ haplotype facilitates sufficiently dense alloantibody deposition for IgG hexamer assembly to occur. These IgG hexamers then direct classical complement activation onto the pulmonary endothelial surface, initiating the excessive leukocyte and platelet responses that have been reported in previous studies to cause acute lung injury in this model (6, 19, 20). Antibody/haplotype combinations in which a single MHC class I antigen is targeted do not enable sufficiently dense antibody binding for IgG hexamer assembly to occur, unless Fc mutations are introduced into the antibody to increase hexamerization. Our findings imply that both lymphocyte crossmatch and single-antigen bead assays for detecting complement-fixing antibodies may lack sensitivity to detect antibodies that activate complement in vivo. The mobility, density, and diversity of antigens on the lymphocytes or solid-phase beads used in these assays do not exactly resemble those on endothelial cell surfaces targeted by donor-specific antibodies in vivo, and our results indicate that each of these factors determined the complement-fixing capability of antibodies. Conversely, antigen density on beads exceeding that found in vivo may give false-positive findings of complement-fixing antibody responses.

By demonstrating that IgG hexamers are important in pathophysiology and represent therapeutic targets in vivo, our work builds on in vitro studies implicating antibody hexamerization in complement activation by antibodies targeting antigens on liposomes, lymphoma cells, or bacterial membranes (15–18, 24, 41). Further clinical translation will require determination of the importance of IgG hexamers in more complex models of diseases involving complement-activating alloantibodies (e.g. AbMR, TRALI, hemolytic transfusion reactions, and hemolytic disease of the fetus and newborn) or autoantibodies (e.g., warm autoimmune hemolytic anemia, antiphospholipid syndrome, myasthenia gravis, and neuromyelitis optica). As SpA-B does not inhibit IgG3-mediated complement activation but IgG3 can assemble into hexamers (15, 16), it will also be important to develop strategies to inhibit IgG3 hexamerization to examine the therapeutic potential of targeting hexamers formed by IgG3 antibodies.

Our results also provide insights into the modes of action of past, present, and potential future therapeutics. Full-length SpA has been used as a therapeutic in the form of a now-discontinued extracorporeal immunoadsorption product (Prosorba column). SpA immunoadsorption efficacy has been observed in patients with symptoms unchanged by plasma exchange, an effect ascribed to leakage of SpA from columns into the bloodstream of patients, resulting in B cell depletion caused by the action of SpA as a B cell receptor superagonist (42). Purified SpA infusions (PRTX-100) were subsequently studied in early-stage clinical trials before abandonment for financial reasons (43). Our results suggest that there may be settings in which therapeutics based on the SpA-B subdomain of SpA have efficacy through prevention of complement activation without the risk of adverse effects related to immune complex formation and B cell superagonism caused by Ig polyvalency of full-length SpA. Donor-derived Ig products (e.g., IVIg and SCIg) are currently used to treat antibody-mediated disease flares. Our observation that SCIg reduced injury responses but did not prevent classical complement activation in vivo is concordant with previous studies concluding that Ig therapeutics act on downstream mediators in vitro and in vivo (35, 44). CSL777 is an attractive potential future therapeutic for the treatment of alloantibody-mediated diseases, as it showed efficacy in our models and had a mode of action that would be anticipated to prevent complement activation by both IgM and IgG antibodies as well as pathophysiology resulting from Fcγ receptors (11, 27, 28). CSL777 also does not present problems with the use of donor-derived products related to sourcing, purification, and concentration for injections (27, 45).

In conclusion, this study provides evidence that IgG hexamers can be important triggers of complement-dependent pathophysiology in vivo. Our preclinical studies support further investigation of IgG hexamerization inhibitors and IgG hexamer decoy therapeutics for use in preventing disease states caused by antibodies and complement activation.

## Methods

### Sex as a biological variable.

Male mice were used, as female mice are not susceptible to 34-1-2S–mediated injury (5).

Additional details on methods are provided in the Supplemental Methods.

### Animals.

B6.C-*H2^d^*/bByJ (B6.*H2^d^*) mice (catalog no. no. 000359) (46) and B6(Cg)-*C1qa^tm1d(EUCOMM)Wtsi^*/TennJ (*C1qa^–/–^*) mice (catalog no. no. 031675) (22) originated from The Jackson Laboratory. B6-background mice were bred with B6.*H2^d^* mice, and their progeny were crossed to produce mice with homozygous expression of H2^d^ MHC antigens for use in experiments (6). B6.ConK^d^-on and B6.Tg(CD2-Tcra,-Tcrb)75Bucy (TCR75) mice were provided by James Zimring (University of Virginia, Charlottesville, Virginia, USA) (21, 47). BALB/c mice were purchased from Charles River Laboratories (catalog no. no. 028). B6;SJL-Tg(FCGR2A)11Mkz/J mice (expressing human FCGR2A isoform R131, The Jackson Laboratory, catalog no. no. 003542) (29) were backcrossed with B6 congenicity (48). Mice were studied at 8–16 weeks of age after maintenance in the UCSF specific pathogen–free animal facility for at least 2 weeks.

### Surface plasmon resonance.

Binding affinities were determined by injecting serial dilutions (0.5–200 nM) of MHC class I monomers (MBL International, catalog no.: TB-5001-M [K^b^ presenting SIINFEKL]; TB-5008-M [D^b^ presenting RAHYNIVTF]; TB-M552-M [K^d^ presenting VYLKTNVFL]; TB-M536-M [D^d^ presenting IGPGRAFYA]; TB-M521-M [L^d^ presenting SPSYVYHQF]) over mAbs bound via amine coupling to SensEye G Easy2Spot sensors (Ssens catalog no. 1-09-04-006), and were assayed in triplicate with an IBIS MX96 surface plasmon resonance (SPR) imager.

### Antibody-mediated acute lung injury model.

As previously described, mice were given i.p. injections of LPS (MilliporeSigma, catalog no. L2880, 0.1 mg/kg) for the “priming” needed to render barrier-housed mice responsive to antibody injections (19). Twenty-four hours after LPS priming, mice were anesthetized (60 mg/kg ketamine plus 40 mg/kg xylazine i.p.), and the indicated antibody treatments were injected into the jugular vein over the course of 1 minute (at 1 mg/kg unless otherwise stated). Antibodies were from Bio X Cell (mIgG2a isotype control clone C1.18.4, catalog no. BE0085; anti–MHC class I clone 34-1-2S, catalog no. BE0180; hIgG1 isotype control, catalog no. BE0297) or newly produced (described below). Lung vascular permeability was measured by giving each mouse 0.01 KBq ^131^I-conjugated albumin (Iso-Tex Diagnostics, NDC:50914-7731) together with i.v. antibody injections, with collection of lungs and blood samples 2 hours later or at cessation of breathing for radioactivity measurements used to quantity the volume of extravasated plasma in lungs (lung vascular permeability). Wet-dry weight ratios of lungs and blood were used to calculate excess lung water volumes (6).

### Immunofluorescence imaging.

Cryosections were made at 200 μm or 400 μm thickness from lungs fixed by inflation with and immersion in 1% formaldehyde in PBS, as previously described (6). Sections were incubated overnight with antibodies targeting C3b/d (clone 11H9, Novus, catalog no. NB200-540), C1qa (clone 4.8, Abcam, catalog no. ab182451), C4b/d (clone 16D2, Novus, catalog no. NB200-541), Scgb1a1 (MilliporeSigma, catalog no. ABS1673), CD41 (clone MWReg30, BioLegend, catalog no. 133902), or S100a8 (R&D Systems, catalog no. AF3059) together with a FITC-conjugated antibody raised against Acta2 (clone 1A4, MilliporeSigma, catalog no. F3777), all at 1:500 with 5% normal donkey serum, 0.1% BSA, and 0.3% Triton X-100 in PBS. After washing, Cy3 or Alexa Fluor 647–conjugated, cross-adsorbed polyclonal secondary antibodies targeting rat, rabbit, goat, and/or mouse IgG (Jackson ImmunoResearch, catalog no.s 712-165-153, 711-165-152, 705-605-147, and 115-605-206) were incubated with sections at 1:500 in PBS plus 0.3% Triton X-100 overnight. After additional washes, sections were either mounted in Vectashield (Vector Laboratories, catalog no. H-1700) for standard confocal imaging on a Nikon A1r microscope, or cleared after staining using the EZ clear protocol (49) for 3D imaging with a Nikon AZ100M confocal microscope.

### Antibody carbamylation.

Carbamylation of 34-1-2S was achieved by incubating 1 mg antibody in PBS plus 0.1 M potassium cyanate (KOCN) for 1 hour at 37°C before buffer exchange back into PBS (24). Control 34-1-2S was subjected to the same process with omission of KOCN.

### Antibody sequencing and engineering.

The 34-1-2S hybridoma was purchased from the American Type Culture Collection (ATCC) (catalog no. HB-79). To generate the Kd1 hybridoma, B6 mice were injected i.v. with 3 × 10^6^ CD4^+^ T cells from TCR75 mice transgenic for a T cell receptor (TCR) specific for a peptide from K^d^ presented by IA^b^, and i.p. with 5 × 10^6^ Con-K^d^-on splenocytes, resulting in an extreme B cell antibody response directed at an immunodominant peptide from K^d^, the only mismatched antigen between donor and recipient. Three days after a boost with an additional i.p. injection of Con-K^d^-on splenocytes, splenocytes from the sensitized recipient were fused with a myeloma cell line as previously described (50), and mAbs specific for K^d^ were identified using Con-K^d^-on splenocytes as targets (21).

Aliquots of mAbs were digested with either peptidyl-Asp metalloendopeptidase, chymotrypsin, elastase, trypsin, or pepsin enzymes. Peptides were then assayed using liquid chromatography coupled to tandem mass spectrometry for sequencing of variable fragments (Bioinformatics Solutions). The amino acid sequences determined were, for 34-1-2S: EVQLQQSGAEFVRPGASVKLSCTASGFNLKDDYMFWVKQRPEQGLEWIGWIAPDNGDTEYASKFQGKATITADTSSNTAYVQLSSLTSEDTAVYYCTTWGYYSYVNYWGQGTTLTVSS (heavy-chain variable region) and DIQMTQSPSSLSASLGERVSLTCRASQDIGSNLNWLQQEPDGTIKRLIYATYSLDSGVPKRFSGSRSGSDYSLTISSLESEDFVDYYCLQYASSPYTFGGGTKLEIK (light chain variable region); and for Kd1: EVLLVESGGDLVKPGGSLKLSCAASGFTFRTYAMSWVRQTPEKRLEWVATIGDDGSYTFYPDNVKGRFTISRDNAKNNLYLQMRHLKSEDTAIYFCARDGLFAYWGQGTLVTVSA (heavy chain variable region) and DIQMTQSPSSLSASLGGKVTITCKASQDIKKNIAWYQYKPGKGPRLLIHYTSTLQPGISSRFSGSGSGRDYSFSISNLEPEDIATYYCLQYDSLLYTFGGGTKLEIK (light-chain variable region). Correct identification of variable domains was confirmed by performing sequencing of the products of 5′ rapid amplification of cDNA ends (5′-RACE) for heavy- and light-chain mRNA using RNA isolated from hybridomas. The isolated 5′-RACE amplicons contained ORFs that encoded the above peptides sequenced by mass spectrometry. These sequences were codon optimized, and antibodies were expressed as chimeric hIgG1 with or without Fc point mutations in a HEK293 cell system and purified using protein A and buffer exchange (Absolute Antibody).

### Pharmacologic treatments.

Recombinant subdomain B from *S. aureus* (SpA-B, amino acid sequence: HHHHHHADNKFNKEQQNAFYEILHLPNLNEEQRNGFIQSLKDDPSQSANLLAEAKKLNDAQAPK, His tag added for purification) was produced in an *E*. *coli* system by GenScript and supplied in protein storage buffer (50 mM Tris-HCl, 150 mM NaCl, 10% glycerol, pH 8.0). At 1–2 hours before i.v. injection, SpA-B (3 mg/kg) or vehicle was mixed with hIgG1-34-1-2S, resulting in a 30% vehicle/70% PBS mixture. Trial formulations of CSL777 (in PBS vehicle), as well as clinical-grade IgPro20 (Hizentra) and the proprietary vehicle for IgPro20, were provided by Rolf Spirig (CSL Behring). Mice were given i.p. injections of CSL777, IgPro20, or the relevant vehicle 1–2 hours before i.v. injections of antibodies at the stated doses.

### Negative stain electron microscopy.

Antibody samples were diluted to 0.01 mg/mL in 25 mM HEPES and 100 mM NaCl and added to carbon-coated grids (TedPella catalog no. 01702-F, manually coated with 20 nm carbon using a Cressington 208 Sputter Coater). Sample-coated grids were stained using 0.75% uranyl formate and imaged on an FEI Tecnai T12 transmission electron microscope.

### Pulse oximetry.

Peripheral blood oxygen saturation (SpO_2_) was measured using a MouseOx+ pulse oximeter with a collar sensor (Starr Life Sciences), with mice breathing room air during recordings. Baseline measurements were taken 1 day after LPS priming. Mice were then given an i.v. injection of hIgG1-34-1-2S under brief isoflurane anesthesia, followed by additional readings taken by averaging the recordings collected over 1-minute sampling periods every 5 minutes over the following 20 minutes.

### Experimental design.

Within-cage randomization was used for group allocations in the studies testing exogenous treatments. Littermate controls from heterozygous crosses were used to test the effect of *C1qa*-KO and *hFCGR2A* expression. Congenic animals housed in the same room were used to study haplotype effects. Handlers were blinded to the group allocations during experiments. Group numbers (*n*) and analytic approaches were predetermined before initiation of the experiments.

### Statistics.

Statistical tests used on each data set and summary statistics plotted on graphs are described in each figure legend. *P* values and multiplicity-adjusted *P* values (*P*_adj._) above 0.0001 are reported in the figures, with asterisks used to highlight *P* values of less than 0.05. Data are presented as the mean ± SEM.

### Software.

GraphPad Prism (GraphPad Software) and InVivoStat were used for graphing and statistical analysis. UCSF ChimeraX was used for molecular graphics (51). Imaris was used to render fluorescence micrographs, and ImageJ (NIH) was used to process electron microscopy data.

### Study approval.

Procedures received ethics approval from the IACUC of UCSF.

### Data availability.

Values for all data points in graphs are reported in the Supporting Data Values file.

## Author contributions

SJC, JCZ, and MRL conceptualized the research. SJC, YS, JJT, NK, DPB, and AEHB contributed to data acquisition and analysis. SJC, MRL, AEHB, GV, ÉB, RS, and JCZ developed methodology. SJC, JCZ, and MRL acquired funding. SJC, JCZ, and MRL wrote the original draft of the manuscript. All authors contributed to the review and editing of the manuscript.

## Supplementary Material

Supplemental data

Supplemental video 1

Supporting data values

## Version 1 03/26/2024

In-Press Preview

## Version 2 06/03/2024

Electronic publication
