# Supplementary material for: IgG hexamers initiate complement-dependent acute lung injury
Source: J Clin Invest. 2024 Mar 26;134(11):e178351. doi: 10.1172/JCI178351 (PMC11142733; doi:10.1172/JCI178351)
Supplement: Supplemental data [file jci-134-178351-s147.pdf]

Supplemental materials and methods

Additional MHC class I antibodies. Additional IgG2a monoclonal antibodies targeting MHC class I antigens used in surface plasmon resonance (SPR) experiments were: clone AF6-88.5.5.3 (BioXCell Cat# BE0121); clone 20-8-4S, produced locally from hybridoma (ATCC Cat# HB-11); clone SF1.1.10 (BioXCell Cat# BE0104); clone 30-5-7S (Thermo Fisher Cat# MA1-70109); clone 34-5-8S (Thermo Fisher Cat# MA1-70108).

Detection of antibody binding to MHC class I antigens using flow cytometry. Spleens of B6.*H2*^{d} mice were dissociated using a syringe plunger and 40 µm cell strainer into 2 ml of PBS + 0.5 mM EDTA. Erythrocytes in this cell suspension were then lysed with addition of red blood cell lysis buffer (20 ml/spleen: 155 mM NH_4Cl, 461 mM $KHCO_3$, 0.1 mM EDTA in deionized H_2O, 5 minutes at room temperature), before resuspension at 1×10^7 cells/ml in 200 µl of FACs buffer (PBS + 0.3% BSA + 0.5 mM EDTA), plus 2.5 µg per sample of Fc block (anti-CD16/32 clone 2.4G2, BioXCell Cat# BE0307). Splenocyte samples were mixed with varying concentrations of carbamylated and non-carbamylated 34-1-2S and incubated at 4 °C for 15 minutes. Samples were then washed 3 times with PBS and stained with anti-CD45-APC (BioLegend Cat# 103112), anti-B220-PerCP (BD Cat# 561086), and anti-mouse IgG-AlexaFluor™546 (Invitrogen Cat# A10036), all at 1:400, as well as DAPI (1.25 µg/ml, Invitrogen Cat# D1306), and then incubated at 4 °C for 20 minutes. Quantification of anti-mouse IgG median fluorescence intensity (MFI) was carried out after gating on live (DAPI-), non-B cell leukocytes ($B220^{low}$, CD45+) using a BD LSRII flow cytometer.

Measurement of platelet hFCGR2A using flow cytometry. Approximately 50 µl of blood was collected from submandibular veins of mice under brief isoflurane anesthesia and immediately mixed with 100 µl acid citrate dextrose anticoagulant (Sigma Aldrich Cat# C3821-50ML). Within 2 hours of collection, 2 µl of blood was mixed with 200 µl of FACS buffer containing anti-CD41-FITC (BD Cat# 5553848) and anti-hFCGR2A-PE (Life Technologies Cat# CD3204) both at 1:400. Expression of hFCGR2A was measured after dilution with ~1 ml of additional PBS and gating on platelets (FSC^{low}, SSC^{low}, CD41+) using a BD LSRII flow cytometer.

Blood platelet counts. Blood collected from the inferior vena cava as part of lung vascular permeability and excess lung water experiments was anticoagulated in Minicollect K2EDTA vials (Greiner Cat# 450480) and assayed using a Genesis hematology analyzer (Oxford Science).

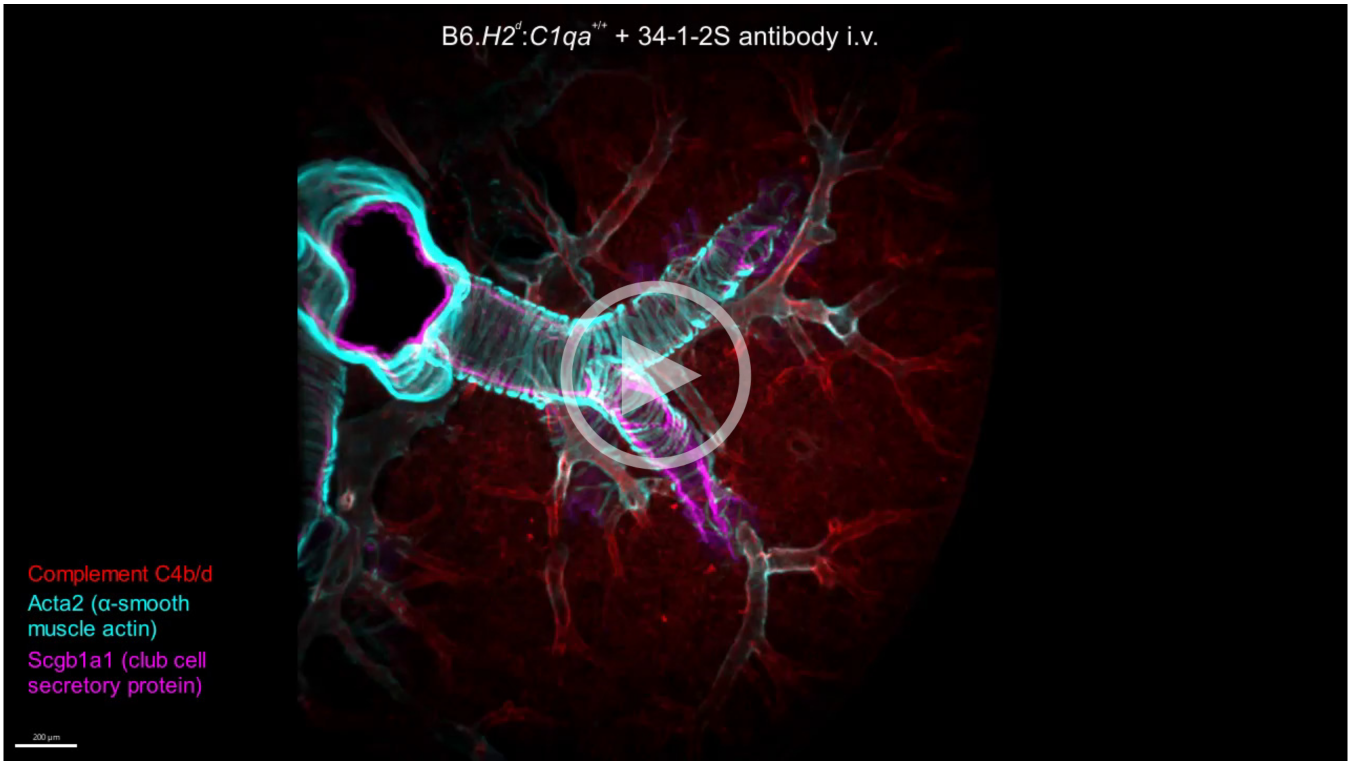

Supplemental Movie 1. Alloantibody-mediated complement C4 split product deposition in the pulmonary vasculature. LPS-primed B6.*H2*^{d} mice of either *C1qa*$^{+/+}$ or *C1qa*$^{-/-}$ genotype were given 34-1-2S or its isotype control i.v. at 1 mg/kg. At 5 minutes (example 1) or 2 hours (examples 2 and 3) after antibody injections, lungs were collected, fixed, sectioned at 400 µm thickness, immunostained and cleared using the EZ clear protocol. Images show staining for complement C4/C4b/C4d (red), Acta2 (α-smooth muscle actin surrounding airways and resistance arterioles, cyan) and in some samples, Scgb1a1 (club cell secretory protein, abundant on epithelium of medium-sized airways, magenta). Detail expansions highlight arterioles that show strong endothelial positivity for complement C4b/d in C1qa-sufficient mice injected with 34-1-2S antibody. Movie file attached as supplementary data.

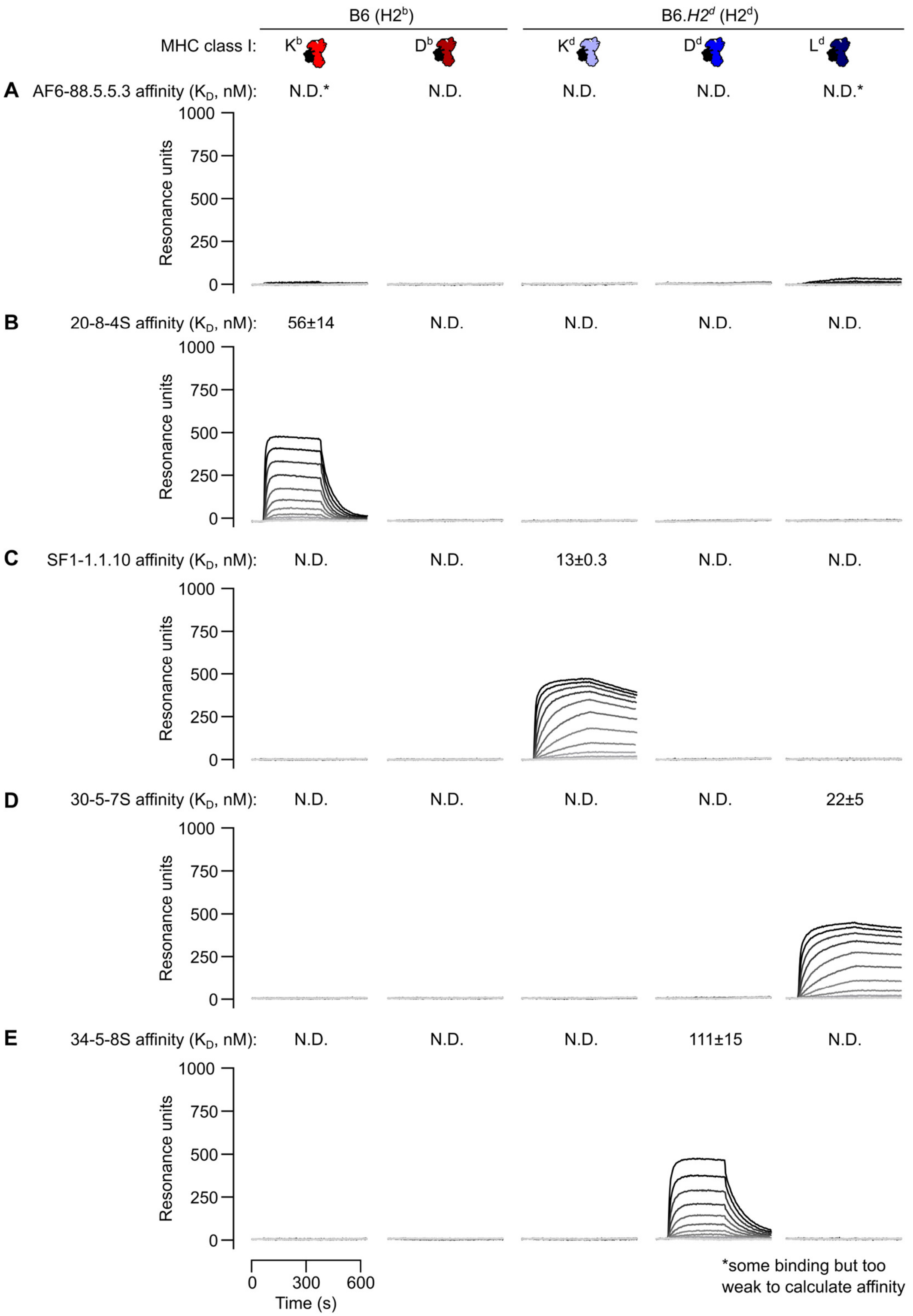

Supplemental Figure 1. Binding of MHC class I monoclonal antibodies to MHC class I monomers. Representative surface plasmon resonance (SPR) sensorgrams showing binding of antibody clones: (**A**) AF6-88.5.5.3; (**B**) 20-8-4S; (**C**) SF1-1.1.10; (**D**) 30-5-7S; and (**E**) 34-5-8S to each of the classical MHC class I antigens expressed by mice with $H2^b$ or $H2^d$ haplotypes. Dissociation constants (K_D) are given as means ± standard deviations (n=3).

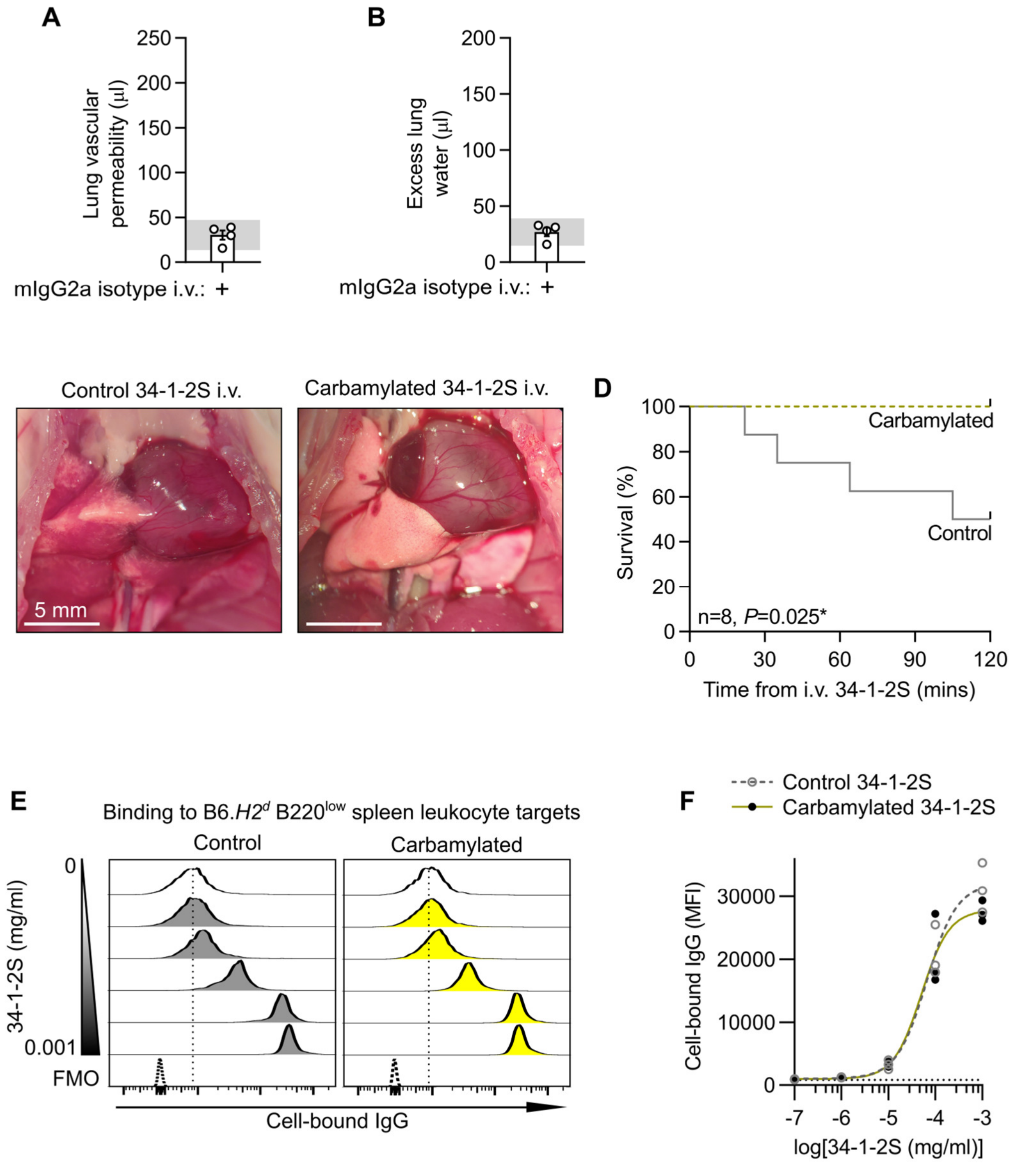

Supplemental Figure 2. Extended characterization of effects of 34-1-2S carbamylation.

(**A**) Lung vascular permeability and (**B**) excess lung water measurements from LPS-primed BALB/c mice measured 2 hours after injection of mIgG2a isotype control (1 mg/kg i.v.), used as 'no injury' controls for data in **Figure 3, C and D**.

(**C**) Gross lung pathology and (**D**) survival data from LPS-primed BALB/c mice given i.v. injections of 1 mg/kg of control 34-1-2S or carbamylated 34-1-2S. (**C**) Shows open-chest views of lungs in situ with visible lung injury in a mouse that developed fatal lung injury after injection of control 34-1-2S, and healthy appearance of lungs after injection of carbamylated 34-1-2S.

(**E**) Representative histograms and (**F**) quantification of median fluorescence intensity (MFI) showing binding of control and carbamylated 34-1-2S to live, CD45+ B220^{low} B6.*H2^{d}* splenocytes assessed by flow cytometry.

Bars and error bars show means ± standard error. Group sizes: (**A** and **B**): n=4 mice; (**C** and **D**): n=8 mice; or (**E** and **F**) n=3 technical replicates. The *P* value reported in (**D**) is from a log-rank test.

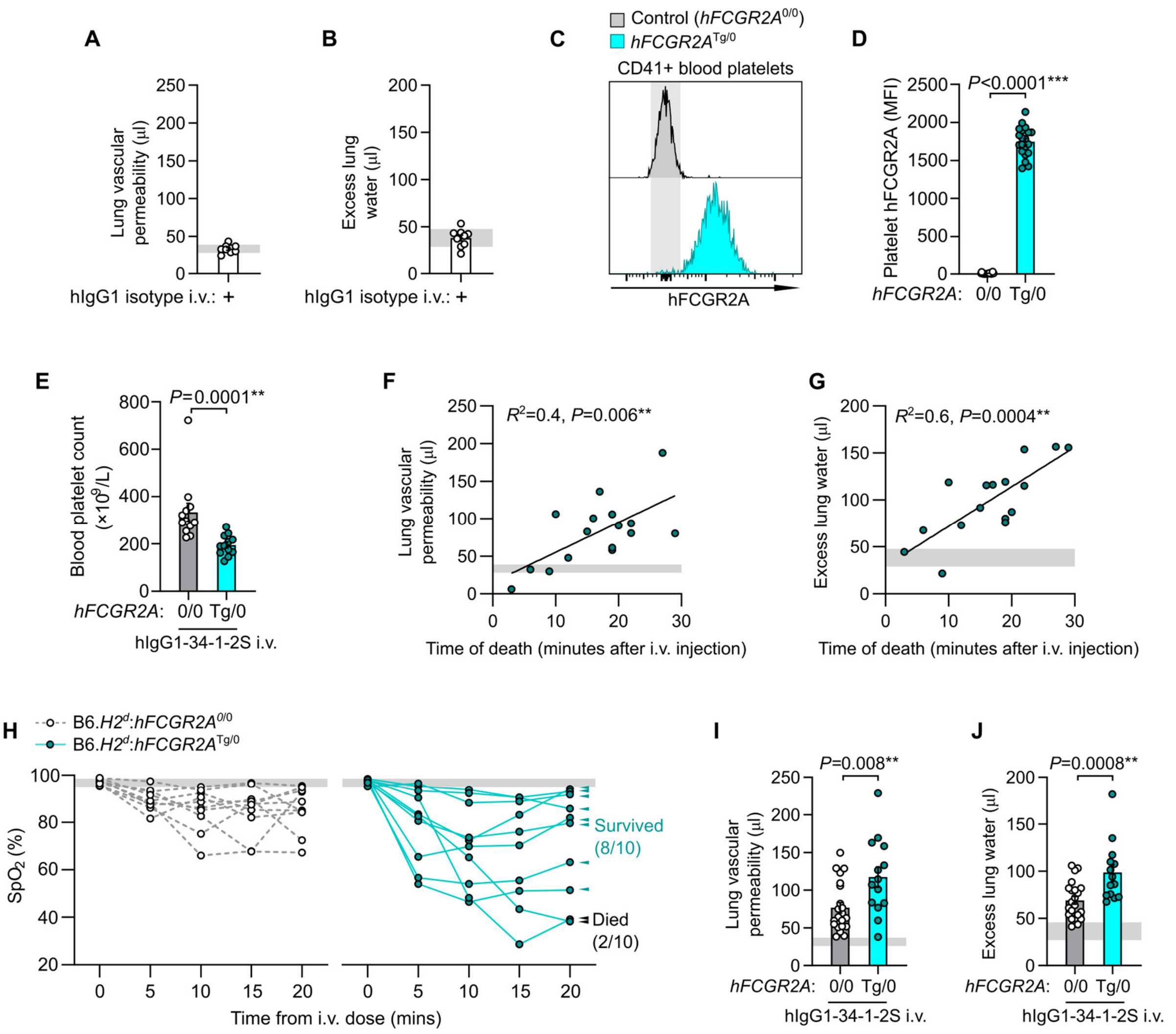

Supplemental Figure 3. Extended characterization of effects of hIgG1-34-1-2S injections and hFCGR2A expression.
(**A**) Lung vascular permeability and (**B**) excess lung water measurements from LPS-primed B6.*H2*^{d} mice measured 2 hours after injection of hIgG1 isotype control (1 mg/kg i.v.), used as 'no injury' controls for data in **Figures 3,4,6, and 7**.
(**C**) Flow cytometry measurements of hFCGR2A expression on platelets from littermates that did or did not inherit the *hFCGR2A* transgene, with median florescence intensity values (MFI) quantified in (**D**).
(**E**) Blood platelet counts from LPS-primed B6.*H2*^{d}:*hFCGR2a*$^{0/0}$ and B6.*H2*^{d}:*hFCGR2a*$^{Tg/0}$ either at 2 hours after hIgG1-34-1-2S i.v. or at time of death.
(**F and G**) Data pooled and re-graphed from non-survivor B6.*H2*^{d}:*hFCGR2a*$^{Tg/0}$ mice in **Figure 6, A-C**, **I-K** and **Figure 7** to show relationship of time of death with extent of lung injury.
(**H**) SpO_2 readings re-graphed from **Figure 6H** to show trajectories of hypoxemia in individual mice.
(**I and J**) Data pooled and re-graphed from surviving B6.*H2*^{d}:*hFCGR2a*$^{0/0}$ and B6.*H2*^{d}:*hFCGR2a*$^{Tg/0}$ mice in **Figure 6, A-C** and **I-K** to demonstrate effect of hFCGR2A expression on readouts given sufficient time for acute lung injury to develop.
Bars and error bars show means and standard errors, group sizes were: (**A** and **B**) n=9, (**D**) n=20, (**E**) n=12, (**F** and **G**) n=16, (**H**) n=10, and (**I** and **J**) n=22, n=14. *P* values are from: (**A**, **B**, **D**, **E**, **I** and **J**) unpaired two-tailed t-tests on log_{10}-transformed data, or (**F** and **G**) F-test for null hypothesis that overall slope of line of best fit is zero.
